# Can Precision Oncology Benefit Patients With Cancers of Unknown Primary?

**DOI:** 10.1093/oncolo/oyad248

**Published:** 2023-09-04

**Authors:** Elie Rassy, Fabrice Andre

**Affiliations:** Gustave Roussy, Départements de Médecine Oncologique, F-94805, Villejuif, France; CESP, INSERM U1018, Université Paris-Saclay, Villejuif, France; Gustave Roussy, Départements de Médecine Oncologique, F-94805, Villejuif, France; Gustave Roussy, INSERM U981, Université Paris-Saclay, Villejuif, France

## Abstract

This editorial calls for a strategic shift in our approach to cancers of unknown primary (CUP), one that generates a comprehensive multidimensional portrait of cancer in patients with CUP, to model the biology at the individual level and identify the right therapeutic target accordingly. The framework of this approach is based on the integration of basic biology, biotechnologies, data sciences, clinical research, and social sciences.

After half a century of advances, cancer research has evolved from a simplistic to a multidimensional approach covering the intricate and dynamic interactions between cancer cell changes that originate within the cells themselves and their surrounding tumor microenvironment.^1^ The alterations occurring within the cancer cells, whether they are genomic, transcriptomic, or epigenetic, play a significant role in carcinogenesis. The complexities of this process account for a substantial biological heterogeneity both within and between metastases occurring in the same organ site or different organ microenvironments.^2^ Furthermore, these complexities are further compounded by the interpatient heterogeneity involving host/microbiota interactions and drug distribution.^3^ The traditional one-size-fits-all approach could not achieve the expected outcomes despite the latest advances in targeted therapies alongside the development of novel therapeutic approaches. The strategic shift toward precision oncology presents an opportunity to integrate the patient’s and tumor’s molecular data to achieve a more comprehensive characterization of the tumor and tailor the therapeutic approach to each patient’s specific needs, ensuring the right treatment, at the right dose, and at the right time.^4,5^

Cancers of unknown primary (CUP) are hard-to-treat cancers where the traditional definition of cancer based on its primary site is not applicable. This entity encloses a diverse group of metastatic tumors without an identifiable anatomical origin at the time of diagnosis, despite extensive investigations.^6^ Some experts regard CUP as an artificial classification, suggesting that it comprises malignant metastases originated from undetected primary tumors, and advocate for thorough diagnostic assessments to identify the primary tumor and tailor-targeted therapy accordingly. Conversely, other experts view CUP as a distinct clinical condition with unique characteristics, negating the need to identify the primary tumor and instead favor the use of CUP-specific treatments.^7^ Advancements in molecular and radiological techniques have dropped the proportion of cancer patients who are diagnosed with CUP to 1%-2% compared to 3%-5% in the early 1990s.^8^ However, this decline may be influenced by oncologists who prefer to consider the patient with an identifiable primary thus allowing him a broader range of innovative therapies. Using this approach, the accuracy of predicting the site-of-origin using (epi)genomic or transcriptomic CUP classifiers and DNA or RNA sequencing ranged between 54% and 98% and around 61% when using deep learning-based algorithms.^7,9^

The management of CUP patients based on a suggested primary offers 2 therapeutic strategies that may sometimes overlap: tumor type-specific therapy and biomarker-guided therapy. However, when employing this straightforward precision medicine approach, treatments tailored to the site of origin did not show a statistically significant improvement in overall survival.^10^ The phase III trial GEFCAPI04 used conventional site-specific therapy based on the suggested primary tumor but encountered several challenges.^11^ One of the main issues was the disproportionate accrual of pancreaticobiliary and advanced squamous cell carcinomas. Moreover, the GEFCAPI 04 trial was presented in 2019, but its design dates back more than a decade ago with site-specific therapy already predetermined in 2011, whereas significant therapeutic improvements have been achieved in the interim. For instance, lung and renal carcinomas of unknown primary appear to benefit from site-specific therapy.^12^

Our review of the literature has revealed that despite the advancements in molecular biology, the recognition of the molecular abnormalities involved in CUP, as well as the identification of the tissue of origin, remain unresolved issues.^13^ Moreover, 3 decades of research were underwhelming and call for a strategic shift in our approach to CUP. To address the limitations of previous trials, we have previously proposed 2 trial designs.^14^ The first design involves a master protocol that employs a common CUP classifier to create a homogeneous study population. This approach allows participants to receive treatment options according to the latest innovations within a flexible, adaptive multiarm, multistage trial. Considering the potential challenges in implementing the aforementioned design, we proposed an alternative practical design following a single-arm phase II design to treat patients with site-specific therapy according to their suggested primary tumor site. The outcomes of these trials would then be compared with results published among historical CUP patients who received similar therapies for known primary sites.

We suggest a holistic approach to patients with CUP to enhance patient-centric implementation strategies, accelerate research progress, promote the acceptance and adoption of molecular tools, and personalize drug access. This approach consists of generating a comprehensive multidimensional portrait of cancer in patients with CUP in order to model the biology at the individual level and identify the right therapeutic target, irrespective of the origin of the cancer. This approach requires an integration of basic biology, biotechnologies, data sciences, clinical research, and social sciences (Fig. 1). The framework of this vision is based on an inductive, bottom-up, research approach that involves 3 essential steps: First, translating breakthrough discoveries in basic science into biomarkers of sensitivity to therapies; second, developing technologies that effectively integrate each component of the cancer biology into multidimensional portraits; and finally, advancing this research toward clinical implementation. The implementation of this biology-driven approach to care for patients with CUP relies on cutting-edge biotechnologies, including organoids within the tumor microenvironment spatial biology, analyzed by new mathematical methods, including artificial intelligence to detect targets. The ultimate goal is to integrate all these parameters over the long term, forming patient-specific cancer holograms. Our vision for implementing this strategy involves a nationwide screening of cancer patients through a centralized portal where patients will be orientated according to their disease characteristics.

**Figure 1.**
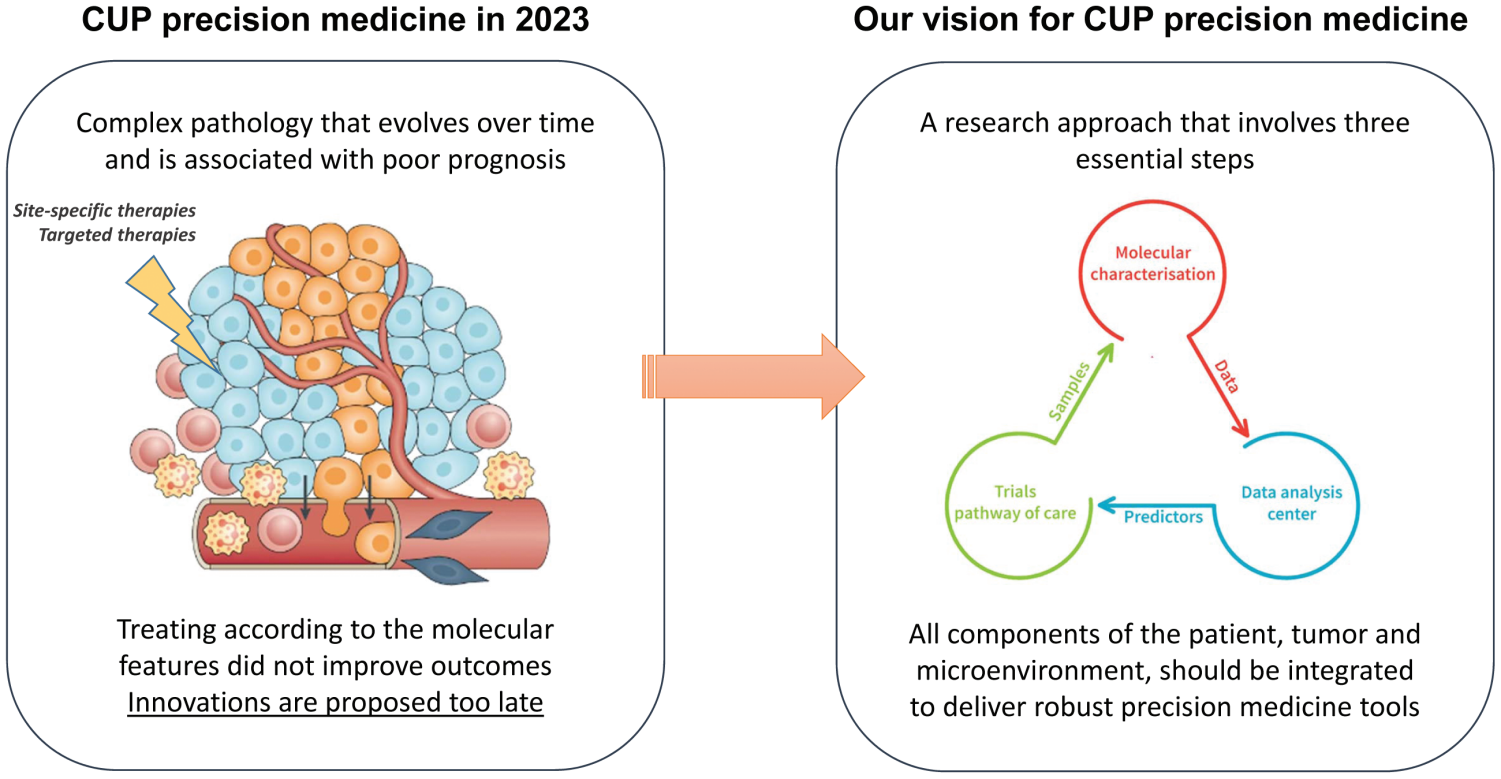
Precision oncology in cancer of unknown primary.

We firmly believe that this second-generation precision oncology centered on biology and irrespective of organ of origin necessitates a cultural shift in our traditional practice, supported by technological advances. The conventional simplistic approach of precision medicine, which consisted of using targeted therapy for a specific target, did not achieve the anticipated results, which is not surprising considering the intricate nature of carcinogenesis, particularly in CUP. This new era requires a redefinition of cancer, extending beyond its organ of origin, and emphasizing the importance of understanding its molecular characteristics within a specific patient at a particular point in time. The success of this vision will not only improve patient outcomes but will also enhance equity in healthcare access and expedite research and access to novel advancements.
